# 90. Late diagnosis of Behçet’s patient with scleritis

**DOI:** 10.1093/rap/rky034.053

**Published:** 2018-09-20

**Authors:** Yasmin Bashir, Qainat Adamjee, C Bernard Colaço

**Affiliations:** 1 Medicine, Imperial College London, London, UNITED KINGDOM; 2 Rheumatology, Central Middlesex Hospital, London, UNITED KINGDOM

**Introduction:** Behçet’s disease (BD) is a systemic inflammatory disorder that was originally described by Hulusi Behçet in 1937, as a triad of recurrent genital and oral ulcers, as well as iritis. BD has often also been referred to as the silk-road disease, due to the usual geographical distribution of the disease along the silk route, with a greater prevalence in Turkey. BD is characterised by recurrent oro-genital ulceration, alongside variable ocular, vascular and pulmonary manifestations. Ocular involvement is seen in around 70% of BD patients and usually occurs after the manifestation of oral and genital ulcers. Only 20% of BD patients experience ocular involvement as the initial manifestation. The most common ocular manifestation is uveitis. However, there are some uncommon ocular manifestations too, including conjunctivitis, episcleritis and scleritis. Scleritis is a severe ocular inflammatory disorder, which has been known to be associated with conditions such as rheumatoid arthritis (RA). However, it is very rarely associated with BD. Although BD is identified using the International Study Group criteria, where the presence of recurrent oral ulcers along with any two of genital ulcers, eye lesions, skin lesions and a positive pathergy test are diagnostic of BD, there is no mention of genetic markers in the criteria. However, HLA-B51 has been shown to be present in over two thirds of BD patients. This case report describes a late diagnosis of BD in a patient who developed scleritis.

**Case description:** YY is a 40 year old male of French origin who presented with episcleritis in his left eye in 2004. This subsequently settled. However, he then developed diffuse anterior/posterior scleritis in his right eye in 2005. He was treated with prednisolone and again things seemed to settle. In 2006, he was diagnosed with reactive right knee arthropathy and was prescribed methotrexate. In 2008, YY relapsed; presented with complaints of photophobia, discomfort and redness in his right eye and was again diagnosed with active scleritis. He was prescribed prednisolone and ketorolac. Although his ocular symptoms seemed to improve, it was noticed that relapsed as soon as he missed his prednisolone tablets. In 2009, he presented to the rheumatology clinic with a rash all over his legs. He seemed very concerned about his right knee, the rash all over his legs and his right eye. It was found that he may be allergic to methotrexate and it was stopped immediately, but this did not improve the rash. In fact, it got worse and spread to his groin and abdomen, as well as to the dorsum of his feet. Blood tests showed leucocytosis, whereas CRP and ESR were normal. With regards to his knee, no definitive diagnosis was made. As he presented with several features of spondyloarthopathy, it was thought he may have psoriatic arthritis. In the meantime, it became a struggle to control the scleritis. YY was given IV cyclophosphamide but his arthropathy did not respond. In 2010, a positive HLA-B51 result was found, which pointed to a possible diagnosis of BD. He was started on thalidomide, followed by infliximab and then rituximab. This seemed to have no effect and he stopped attending clinic. He was then referred to the department in 2017 as a new referral and it was only then that a formal diagnosis of Behçet's-related scleritis was made. Since then his condition is being managed, with efforts to try to wean him off prednisolone and for him to lose weight. He has also commenced a wheat-free diet. With a retrospective look at his history, it was found that 2004, the year he had his first attack of scleritis, was the worst year of his life. Not only did he go through a messy divorce, he also went through a financial crisis. This was extremely stressful and may have been a potential trigger for his BD scleritis.

**Discussion:** Scleritis is a rare ocular manifestation of BD, but it is important that it is not missed. In this case, it wasn’t until a positive HLA-B51 result came through that a definitive diagnosis was made. If this had been made earlier, he could have been treated better and gone through a less stressful time because of his condition.

**Key Learning Points:** HLA-B51 is a key diagnostic tool in the diagnosis of BD. It is important that patientsare screened carefully and that their symptoms are investigated thoroughly to speed up diagnosis. Even if a rare manifestation presents, it should not be ignored. In addition, stress is a key trigger for BD which should always be investigated in such patients to avoid making a late diagnosis.

**Disclosure: Y. Bashir:** None. **Q. Adamjee:** None. **C. Colaço:** None.

